# Simulation of complex data structures for planning of studies with focus on biomarker comparison

**DOI:** 10.1186/s12874-017-0364-y

**Published:** 2017-06-13

**Authors:** Andreas Schulz, Daniela Zöller, Stefan Nickels, Manfred E. Beutel, Maria Blettner, Philipp S. Wild, Harald Binder

**Affiliations:** 1 Preventive Cardiology and Preventive Medicine, Center for Cardiology, University Medical Center of the Johannes Gutenberg-University Mainz, Langenbeckstraße 1, Mainz, 55131 Germany; 2 Institute of Medical Biostatistics, Epidemiology and Informatics, University Medical Center of the Johannes Gutenberg-University Mainz, Obere Zahlbacher Str. 69, Mainz, 55131 Germany; 3 Center for Translational Vascular Biology (CTVB), University Medical Center of the Johannes Gutenberg-University Mainz, Langenbeckstraße 1, Mainz, 55131 Germany; 4 Center for Thrombosis and Hemostasis, University Medical Center of the Johannes Gutenberg-University Mainz, Langenbeckstraße 1, Mainz, 55131 Germany; 5 DZHK (German Center for Cardiovascular Research), partner site RhineMain, Mainz, Langenbeckstraße 1, Mainz, 55131 Germany; 6 Department of Ophthalmology, University Medical Center of the Johannes Gutenberg-University Mainz, Langenbeckstraße 1, Mainz, 55131 Germany; 7 Clinic for Psychosomatic Medicine and Psychotherapy, University Medical Center of the Johannes Gutenberg-University Mainz, Langenbeckstraße 1, Mainz, 55131 Germany; 8 Institute for Medical Biometry and Statistics, Faculty of Medicine and Medical Center - University of Freiburg, Stefan-Meier-Str. 26, Freiburg, 79104 Germany

**Keywords:** Simulation, Planning, Biomarker, Risk prediction, Added value, Random forest, Brier score, AUC, Nagelkerke- *R*^2^

## Abstract

**Background:**

There are a growing number of observational studies that do not only focus on single biomarkers for predicting an outcome event, but address questions in a multivariable setting. For example, when quantifying the added value of new biomarkers in addition to established risk factors, the aim might be to rank several new markers with respect to their prediction performance. This makes it important to consider the marker correlation structure for planning such a study. Because of the complexity, a simulation approach may be required to adequately assess sample size or other aspects, such as the choice of a performance measure.

**Methods:**

In a simulation study based on real data, we investigated how to generate covariates with realistic distributions and what generating model should be used for the outcome, aiming to determine the least amount of information and complexity needed to obtain realistic results. As a basis for the simulation a large epidemiological cohort study, the Gutenberg Health Study was used. The added value of markers was quantified and ranked in subsampling data sets of this population data, and simulation approaches were judged by the quality of the ranking. One of the evaluated approaches, the random forest, requires original data at the individual level. Therefore, also the effect of the size of a pilot study for random forest based simulation was investigated.

**Results:**

We found that simple logistic regression models failed to adequately generate realistic data, even with extensions such as interaction terms or non-linear effects. The random forest approach was seen to be more appropriate for simulation of complex data structures. Pilot studies starting at about 250 observations were seen to provide a reasonable level of information for this approach.

**Conclusions:**

We advise to avoid oversimplified regression models for simulation, in particular when focusing on multivariable research questions. More generally, a simulation should be based on real data for adequately reflecting complex observational data structures, such as found in epidemiological cohort studies.

## Background

When planning a new, potentially large cohort study, simulations can help to judge aspects such as sample size or choice of a statistical approach that might be needed for adequately investigating the effect of biomarkers for an outcome of interest. In particular, such simulation studies allow to take potentially complex correlation structures, covariate distributions and potentially non-linear effects or interactions into account when investigating several biomarkers and known risk factors simultaneously. Therefore, a simulation study may be more adequate for example to assess the sample size needed than using probably oversimplifying sample size formulas. A simulation might also be useful beyond sample size planning, e.g. for picking good measures for biomarker performance. Since a simulation study could also be based on oversimplifying assumptions, at the risk of answers that are not better than, e.g., closed-form sample size formulas, it is of prime importance to use a data generating model of adequate complexity, reflecting realistic data structure. Naturally, this entails the danger of requiring a large amount of information about the population and covariate structure of interest, or introducing a considerable number of simulation parameters that cannot be selected adequately. In the following, this work focusses specifically on how to generate correlated covariates with realistic distribution and on what generating model should be used for a simulated outcome to deal with a complex multivariable research questions, exemplarily considering the task of ranking biomarkers with respect to their added value.

There is a lack of literature on sample size or power calculation methods with consideration of correlated multidimensional covariate data in a regression model and as well on a simulation methodology for this setting. Most of the established methods are using sample size formulas for a regression model with only one covariate [1, 2]. Schmoor et al. [3] proposed a sample size formula for a prognostic problem, where additionally a second correlated factor is considered. This requires knowledge about the joint distribution of these two factors and is restricted to a Cox proportional hazards model with one predictor of interest. Jinks et al. [4] derived a formula for a multivariable prognostic model based on the overall prognostic ability, where the prognostic ability is quantified by the measure of discrimination D and sample size calculation is based on the significance of the D value. Comparable approaches can be applied with the overall discrimination ability (AUC) [5] of the model, for which a sample size calculation can be derived as well [6, 7]. Other authors discuss methods for the problem, where the number of predictors is larger than the actual number of samples. This introduces a selection problem, where informative predictors have to be identified in a mixture of informative and non-informative predictors. De Valpine et al. [8] gave a two-step method, where in the first step a simulation is used to reproduce the selection process of informative predictors and in a second step an approximation method for a linear discriminant problem is used. A similar two step approach was developed by Dobbin et al. [9] but with another methodology. A further approach with a variable selection step was proposed in Götte et al. [10], where the sample size determination is focused on the prediction accuracy instead of power. Unfortunately, most of these approaches are based on uncorrelated variables. Binder et al. [11] investigated different scenarios with a small or large amount of information, different covariates distributions and non-linear functional forms of relationship. The simulation revealed the importance of aspects like covariates distributions or functional form and demonstrated the impact. However, the primary aim of that work was not the planning of new studies but comparing approaches for modeling of non-linear effects. Therefore, the present work specifically investigates the degree of complexity that may be required for a realistic simulation study and techniques to use for an adequate generating model.

We exemplarily consider settings with a binary outcome, which are frequently found in observational data for biomedical research questions considering disease risks. However, most aspects of the simulation method can be easily applied to a continuous outcome as well. As candidate technique for generating simulated covariate distributions, i.e. biomarkers and established predictors, two approaches were compared. Drawing from multivariate normal distributions or additionally transformation according to a known empirical distribution to mimic this distribution as exactly as possible. For generating the clinical outcome, standard linear models and extensions via non-linear terms and interactions were used. As a non-parametric approach the random forest model was considered, which requires individual data as basis. As gold standard, repeated sampling from a large population-based, epidemiological cohort, the Gutenberg Health Study (GHS) was used, and judged how close simulated data based on aggregate information, such as odds ratios or correlation matrices (which might be found e.g. in the literature), agree with the gold standard. The use of a pilot study [12] as basis for simulation was also considered, and the effect of the pilot study size was investigated. As a measure to assess the performance of simulation compared to the defined gold standard, the ranking of biomarkers based on simulated data and based on repeated draws from the population data, were compared. For ranking biomarkers according to their added value, the difference in Brier score, the increase in AUC and the difference in pseudo- *R* ^2^ were considered as added value measures.

In “Population sample” section the GHS study and the exemplary biomarkers and endpoints to be used for investigating simulation approaches are introduced. Concerning the latter, the overall simulation structure is presented in “The general simulation structure” and discuss simulation of covariates in “Covariate matrix” section, and different approaches for generating a simulated phenotype in “Clinical response generating” section. Different measures for added value are discussed in “Quantifying added value” section and the simulation quality criterion in “Reference ranking” section. The population results are presented in “Population sample resultsPopulation sam-ple results” section. Results on different strategies for simulation are presented in “Reference mean ranks” and in “Comparison of simulation approaches” sections. “Pilot sample size” section specifically investigates different pilot study sizes. Concluding remarks are given in “Discussion and Conclusions” sections.

## Methods

### Population sample

As an application example, the Gutenberg Health Study (GHS [13]) sample was used. The GHS is a population-based prospective, observational, single-center cohort study from Germany at the University Medical Center in Mainz. With the first 5000 participants enrolled from April 2007 to October 2008, it is so far a cross sectional, large, population based sample. The primary aim of the GHS study is to evaluate and improve cardiovascular risk prediction. The participants are aged between 35 and 74 years with nearly equal proportion of men and women. The sample was taken from the population in Mainz and Mainz-Bingen area in Germany. The whole study sample includes 15010 individuals. The analysis was restricted on the first 5000 enrolled participants due to the fact that the measurement of the biomarkers of interest was only accomplished for this subsample. After quality control and data cleaning using the complete case principle for the variables of interest, a sample with 4519 individuals remains. Most missing values occurred in the outcome. Missing values were randomly distributed and resulted mainly from logistical problems. Specifically, the binary variable functional cardiac disorder (FCD [14]) was used as medical outcome. The focus of this work was on a binary outcome, because this approach is commonly used in medicine and plays a more important role in risk prediction than continuous traits do. A basic prediction model for this event was defined as a simple model with sex, age and body-mass-index as covariates. This basic model was extended with different biomarkers. One biomarker was added at a time, and the improvement in prediction was evaluated with three different added value methods described in Quantifying added value. The following biomarkers of interest were selected in advance: MR-proADM, Nt-proBNP, hs-CRP, CT-proAVP and MR-proANP. The results in GHS sample are presented in more detail in 8.

### The general simulation structure

**Fig. 1:**
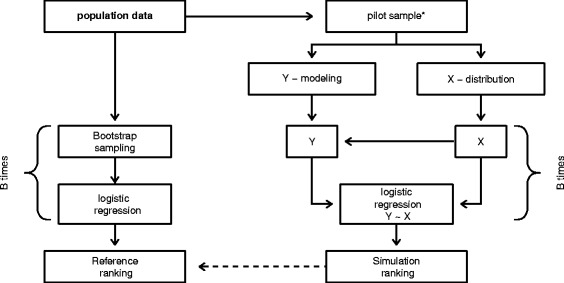
Structure of the simulation. On the *left side* the determination of reference values is illustrated and on the *right side* the structure of the simulation procedure. *This step is skipped for the first part of simulation where the best simulation model was investigated and only used to determine the needed sample size of a pilot study

For exploring the best simulation approach, the following simulation structure was used. The generation algorithm of artificial data can be divided into two parts. First, the covariate data set which included established predictors and all biomarkers of interest need to be generated based on population data. In this regard, the distribution of simulated covariates, including the correlation structure, should reflect the structure in the real data. Two different approaches were used in general for this, the covariates either follow a multivariate normal distribution and or an empirical distribution extracted from existing data, e.g. a pilot study. To evaluate and illustrate the approach, the population data that cover non-normal data distributions was used. Two approaches are described in more detail in 8. The second part is the generation of a simulated clinical response using the simulated covariates of the first part. For this purpose, four different approaches with increasing complexity were used: starting with a simple logistic regression model, followed by a logistic regression model which includes selected interaction terms, and a generalized additive model (GAM) model with non-linear effects. The last approach was the random forest model, a rather complex approach. All approaches are described further in 8. In total, four different approaches for the clinical response simulation and two different methods for covariate data generation were investigated. To compare simulation approaches, a gold standard is required on which the evaluation of the simulation quality can be judged. This gold standard has to be reproduced with simulated data. As a gold standard, the reference ranking of biomarkers and the reference values of the added value measures in application example were used. The application example is described with more details in 8 and the ranking procedure is described in 8. The four approaches were compared without the consideration of the pilot study size. This means, for the generating of artificial data, the whole population sample was taken as a source of information. The pilot study size will be investigated at the end for the best simulation approach. The simulation design is presented schematically in Fig. 1: on the left side the determination of reference values is displayed and on the right side the structure of the simulation procedure [15]. All simulations were done in R version 3.2.2 (2015-08-14) [16]. Additionally the following R packages were used: mvtnorm version 1.0-3 [17, 18], gam version 1.12 and randomForest version 4.6-12 [19].

### Generating artificial data

#### Covariate matrix

For a successful simulation of a realistic sample, covariate data need to be simulated in a sufficient way. One of the major aspects for the simulation is the correlation structure of the covariate matrix. Not only the correlation between markers and basic model covariates is important, the correlation between markers themselves has a key role. Consequently, the whole correlation matrix including all markers of interest and known risk factors must be taken into account. A natural way to simulate the distribution of covariates while simultaneously considering a correlation structure is to use a multivariate normal distribution based on the correlation matrix, location and dispersion of real data. Where a predefined covariance matrix can be given to determine the variance as well as the correlation structure of generated data [20], this covariance matrix can be easily obtained from the population data. Since the dichotomous variable sex was included into the model, all random covariate data were generated sex-specifically and pooled afterwards. As all covariates of interest, except sex, were nearly normally distributed or log-transformed to approximate the normal distribution. Though the method covers the correlation structure of the original data well, variables are rarely exact normally distributed in a real data set. A more exact method to mimic the real distribution is to generate the covariate matrix with multivariate normal distribution and to take the corresponding quantiles from the empirical distribution of a real data set. It requires more information, but reflects even small deviations from the normal distribution if present, without destroying the correlation structure. By using this method, artificial data with a correct correlation structure can be generated that perfectly mimic the true distribution. This can be seen as the most realistic and sophisticated simulation method of the covariate data. In the simulation of this work, both approaches and the benefit of these additional efforts were explored.

#### Clinical response generating

For the generation of the clinical response, the relationship between outcome and the covariate matrix has to be taken into account as accurate as possible. For this purpose, prediction models for the outcome were fitted in population data using different modeling approaches. These prediction models are then used to predict the probabilities of the event given the artificial, simulated covariate data. To generate the outcome for the simulation data set, random numbers were generated from the binomial distribution given the predicted probabilities. Even if the biomarker comparison is made by a simple logistic regression model, more complex models can be used for the simulation of the relationship. Additionally, in terms of comparison of markers it is essential that the association to the outcome is simulated considering all markers simultaneously. For that purpose, the prediction models for the binary endpoint in the population data were fitted using all markers and covariates in one model. For the simulation, a set of different models with rising complexity were selected. The simplest approach would be the modeling of the relationship in a linear way with a logistic regression (GLM). Additional interaction effects can be taken in account, which leads to a GLM model with interaction terms (GLM+I). For this model, a set of the six strongest pairwise interaction effects with a stepwise bidirectional selection method based on AIC (Akaike information criterion) [21] was selected and added to the GLM model. Following interactions were selected automatically: sex with age, CT-proAVP with age, Nt-proBNP with MR-proANP and interactions of MR-proADM with BMI, Nt-proBNP, CT-proAVP and MR-proANP. Where even not significant biomarkers were included in interactions and seem to improve the global model. The resulting information could be important because the interactions between the markers cannot be detected in a prediction model including only a single marker, but this may influence the overall results of the simulation. If the relationship between the outcome and the biomarkers is in reality a non-linear relationship, a non-linear modeling would be more appropriate. Generalized additive model with smoothing splines (GAM) [22] was used to model complex non-linear relationships if present, but interactions are omitted in this approach. To cover non-linear relationships and complex interaction structures simultaneously, a more complex model could be necessary. One possibility is to use the random forest model (RF) [23], based on classification trees (CART) [24–26]. For the random forest models, no pruning step was performed, so all trees were maximal grown trees. The number of trees was set to 1000 and the number of variables randomly sampled as candidates at each split was $\left \lceil \sqrt {p}\right \rceil $ [27], where *p* is the number of predictors available. This method is described in more details in the next section 8. In this work only both methods to generate the covariate data with the GLM and random forest approaches are presented. For all other approaches, only covariate data simulated based on the empirical distribution are presented as this leads to better simulation results.

#### Random Forest approach

Since random forest (RF) plays an important role in the simulation and is not a standard method, it is described in more detail in the following. A short overview of the construction of RF can be found at the end of the section. Random forest is an ensemble of classification or regression trees (CARTs). For the case of a binary outcome, the classification trees are used. The trees in RF are unpruned. This means that each tree is grown to the largest extent possible, but may require a minimum node size of terminal nodes, usually 1. Each tree is grown in a bootstrap subsample drawn from the original training sample. Let *N* be the number of individuals in the whole training sample and *N* ^∗^ the size of a bootstrap sample. In usual, *N* ^∗^=*N* for sampling with replacement and *N* ^∗^<*N* for sampling without replacement. For the simulation, sampling with replacement was used. The number of independent individuals in the bootstrap sample is then $\acute {N^{*}} \approx 0.632\cdot N$, see [28]. The remaining $N-\acute {N}^{*}$ individuals are out-of-bag (OOD) and can be used for an internal validation or out-of-bag prediction, which is not explained here further. One of the tuning parameters of RF is the number of trees to be generated. Let *B* be the number of trees, and consequently the number of bootstrap samples in RF, often also called *ntree*. Another source of diversity in RF is the fact that not all predictor variables are used at the same time, rather a set of randomly selected predictors is used in each node for split in a tree. Let *m* identify the total number of predictors available in the training sample and *mtry* the number of predictors randomly chosen in each node. Consequently, *mtry* is another tuning parameter of RF. The default for classification problems is usually $\left \lceil \sqrt {m}\right \rceil $. Classification trees use a splitting function called Gini-index to determine which attribute to split on and what the best cutoff is. Gini-index is defined as *G*_*k*_=2*f*(1−*f*), where *f* represents the fraction of events assigned to node *k*. In contrast to using one classification tree, RF returns not only the classification decision but can also estimate the predicted probability for an event. For *B* trees in RF, the predicted probability for a new individual is:

$$\hat{P}(y=1|\mathbf{x})=\frac{1}{B}\sum_{b=1}^{B}\pi_{b}(\mathbf{x}), $$

where *π*_*b*_(**x**) is the majority vote in terminal node where the new individual is dropped in for *b*th tree, so the classification decision of a single tree for outcome status *y*∈{0,1}, given the covariate matrix **x**. For more information and features of RF see [29, 30].

The construction of RF is described in the following steps:

1. Select randomly a total of *N* ^∗^ individuals from the original training sample, with replacement. This leads to a bootstrap sample. Repeat this procedure *B* times.
2. In each bootstrap sample, grow an unpruned classification tree. The tree is constructed by recursively splitting data into two distinct sub-samples. At each node, randomly select *mtry* predictors from the total *m* predictor variables. Choose the best split from among the *mtry* predictors by minimize the Gini-index as a measure of node purity.
3. For calculation of predicted probability, each new individual is dropped down a tree until its terminal node. The majority voting for an event status in this terminal node is determined. The probability estimate is then the average of majority votes over all trees.

### Gold standard

#### Quantifying added value

For comparing the predictive strength of biomarkers, the concept of added value that describes the prediction performance of a model was chosen. This can be measured with several different established measurements. Three of them without intend to have a complete list of existing measures were selected. The first one, the Brier score [31, 32], measures the mean squared difference between the predicted probability and the actual outcome. It takes values between zero and one, since this is the largest possible difference between a predicted probability and a binary outcome. The lower the Brier score, the better the prediction performance. The Brier score is defined for a binary outcome as $BS=\frac {1}{n}\sum _{i=1}^{n}(p_{i}-y_{i})^{2}$, where *p* is the predicted probability, *n* is the sample size and *y* is the actual, observed outcome. The second common measure is the area under the curve AUC [5, 7, 32–34] from Receiver Operating Characteristic (ROC) methodology which quantifies the discrimination ability. It can be interpreted as the probability that a randomly selected subject with an event will be ranked higher in terms of predicted probability than a randomly selected subject without an event. One possible definition of AUC is given by $AUC=\frac {1}{n_{1}n_{0}}\left (\sum _{i=1}^{n}(rank(p_{i})y_{i}) - \frac {n_{1}^{2} + n_{1}}{2}\right)$, where *n*_1_ is number of events and *n*_0_ is the number of non-events. Third, the coefficient of determination *R* ^2^, in this case for a binary outcome the generalization of *R* ^2^ for generalized linear models from Nagelkerke [35] was used. Nagelkerke *R* ^2^ coefficient is scaled to a minimum of 0 for no determination and a maximum of 1 for perfect determination. The definition of *R* ^2^ with log-likelihood is $\frac {1}{e^{(-2LL_{0}/n)}-1} \left (e^{((-2LL_{1} + 2LL_{0})/n))}-1\right)$ where the *L* *L*_0_ is the log-likelihood from the null model only with the intercept term and *L* *L*_1_ is the log-likelihood from the model of interest.

Since to quantify the improvement of the extended model, including an additional marker, compared to the basic model, it is straightforward to use the difference in these measures. This results in following three measures: Brier score difference that has the form

$$BSD=\frac{1}{n}\left(\sum_{i=1}^{n}\left(p_{1i}-y_{i}\right)^{2}-\sum_{i=1}^{n}\left(p_{0i}-y_{i}\right)^{2}\right), $$

the *p*_1_ stands for the predicted probability from the model with the new marker and *p*_0_ for the predicted probability from basic model. The increase in AUC could be reduced to the form

$$\begin{array}{@{}rcl@{}} IAUC=\frac{1}{n_{1}n_{0}}\left(\sum_{i=1}^{n}\left(rank(p_{1i})y_{i}\right) - \sum_{i=1}^{n}\left(rank\left(p_{0i}\right)y_{i}\right)\right). \end{array} $$

Nagelkerke *R* ^2^ difference,

$$R^{2}D=\frac{1}{e^{(-2LL_{0}/n)}-1} \left(e^{(-2LL_{1}/n)}-e^{(-2LL_{2}/n)}\right), $$

with *L* *L*_1_ as log-likelihood from the basic model and *L* *L*_2_ from the extended model. The different measures represent different aspects of improvement in prediction, like calibration for Brier score or discrimination ability for AUC [36]. This small set of measures is a good representation of most common performance measures with different approaches and covers the most important aspects of added value.

#### Reference ranking

As a criterion for simulation success, the relative ranking of biomarkers was used. It reflects the biomarker comparison study aims in a direct and intuitive way and allows the comparison of the results from different added value measures. Therefore, the top three markers were ranked by each added value measure separately. The simulation is restricted to the top three markers because the others have very small to non-existent effects, see 8. As the basic prediction model is used as a reference for all markers, ranking based on the added value measure itself or on the difference of it lead to the same ranking. To get reference rankings, a resampling method on population data was used, in this case the target criterion is the mean rank of markers. To access the mean rank, 10000 bootstrap samples from population data were drawn. By bootstrapping with real data the distribution and correlation structure of population data is considered in a natural way. For each bootstrap sample, the added value measures were calculated and compared between each biomarker. This leads to a specific rank in each bootstrap sample for each of the top three markers. The mean rank of a marker from all bootstrap samples is used as the reference. This reference has to be replicated in the artificial data in the simulation to ensure the applicability of the simulation approach. The resulting reference rankings are shown in 8. Additionally, the similarity of absolute added values from simulation results to reference values as a more specific criterion was examined. A good consistency in absolute values would demonstrate even better simulation accuracy.

## Results

### Population sample results

To ensure a stable and well specified model for risk prediction, model diagnostics were carried out in the population data for all models, including the basic model, the extended model with one biomarker and a full model with all markers simultaneously. Model diagnostic covers calibration, influential observations and collinearity. The event frequency was 26.7*%* (*n*=1205 *o* *f* *N*=4519), so even in a full model there were 150 events per covariate. The overall calibration was good in all models as the mean predicted probability ranged between 26.02% and 26.87%. The calibration in subgroups of prediction was similar good in all models and only led to a significant Hosmer-Lemeshow test [37] (*p*=0.042) in one model, which shouldn’t be over-interpreted with this large sample size. By using the Cook’s distance [38, 39], no strong influential observations were detected. The Cook’s distanced is based on a single case deletion statistic which quantifies the changes of estimates after removing a single observation.

The variance inflation factor (VIF) [40, 41] was used to examine the collinearity. Values of the VIF greater than 5 were considered as problematic. In the single extended models with only one additional marker, no VIF values over 2 were observed. In the full model with all biomarkers incorporated simultaneously, the maximum VIF was 2.6 for MR-proANP. In summary, the basic model was stable and well calibrated; there were no collinearity problems and no strong outliers or influential observations after the log-transformation of markers.

Regarding the prediction ability of the model with sex, age and body-mass-index as covariates, the basic model had a moderate predictive value according to the three measures of added value. The AUC of the basic model was 0.76, the Brier score was 0.163 and the *R* ^2^ 0.227. A basic AUC of 0.76 is in mid-range between random (*A* *U* *C*=0.5) and perfect discrimination (*A* *U* *C*=1). Consequently, the basic prediction model was reasonable, but offers enough space for improvement in prediction. Nevertheless, the evaluation of the models additionally incorporating one of the biomarkers of interest yielded in only a weak influence of the biomarkers. The associations with MR-proADM, Nt-proBNP and hs-CRP, respectively, was significant on the 5% level and the association with CT-proAVP and MR-proANP, respectively, was not significant. All results can be seen in Table 1. The top three markers were clearly arranged by strength in prediction improvement with all three added value measures. The improvement by including the markers in the model remains under the expectations and provides only small differences. As CT-proAVP and MR-proANP were non-informative, the simulation was restricted to the top three markers, MR-proADM, Nt-proBNP and hs-CRP. Additionally, the effect of hs-CRP is very weak and doesn’t look very promising, but it can be used for comparison with the other top two markers.

**Table 1:** Logistic regression models: results from population data

Marker	OR per-SD	Lower 95%CI	Upper 95%CI	*p*-value	Brier score diff.	Increase in AUC	*R* ^2^ difference
MR-proADM	1.18	1.08	1.30	0.00035	-0.00048	0.00232	0.00349
Nt-proBNP	1.15	1.05	1.25	0.0017	-0.00033	0.00151	0.00272
hs-CRP	1.08	1.00	1.17	0.044	-0.00024	0.00084	0.00111
CT-proAVP	1.04	0.96	1.12	0.36	0.00000	0.00014	0.00022
MR-proANP	0.99	0.91	1.07	0.75	0.00000	-0.00004	0.00003

All biomarkers were log-transformed. The models were adjusted for sex, age and bmi. The basic model had Brier score of 0.163, AUC of 0.759 and *R* ^2^ of 0.227

### Reference mean ranks

After evaluating the added value measures in bootstrap samples from population data, these values were used to obtain the reference ranking. The data is presented in Table 2 as mean ranks.

**Table 2:** Mean ranks

Ranking measure	MR-proADM	Nt-proBNP	hs-CRP
Brier score difference	1.57	1.99	2.43
Increase in AUC	1.38	1.96	2.66
*R* ^2^ difference	1.45	1.86	2.69

Mean rank is calculated on 10000 bootstrap samples from population data. Only the top three markers were ranked

The mean rank for MR-proADM ranges from 1.38 using AUC to 1.57 using the Brier score. Using the *R* ^2^ resulted in a mean rank of 1.45, which lies somewhere in between. Consequently, the AUC shows a stronger ability to separate the top marker than the Brier score or the *R* ^2^ in the population data. For Nt-proBNP, Brier score and AUC were about 2, the *R* ^2^ provided a little smaller mean rank of 1.86 and thus a little higher ranking. The mean rank of the third marker is not important, because it’s already completely determined with the first two ranking positions. It is not exactly clear how the differences can be explained. One possibility could be the fact that different measures represent different aspects of prediction like discrimination or calibration. Another source could be small deviations from the assumptions like linearity or normal distribution, with a heterogeneous effect on the different measurements. The second explanation would make it even more important to cover these aspects in the simulation.

### Comparison of simulation approaches

#### Mean ranks criterion

**Fig. 2:**
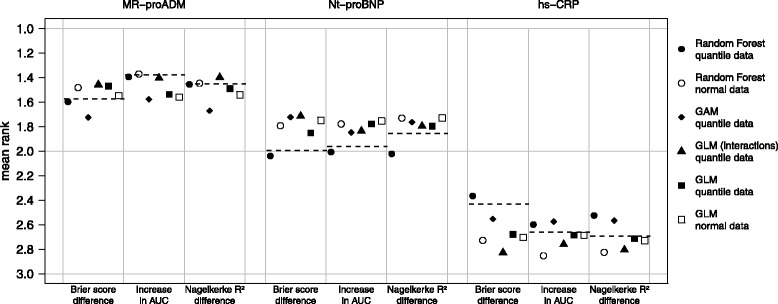
Simulated mean ranks. The mean rank is based on 10000 simulation runs, with different methods of data generation. The *dashed line* represents the reference mean ranks from population data. *Normal data*: multivariate normal distributed covariate data. *Quantile data*: covariate data drawn from the empirical distribution. *GLM*: modeling of the relationship with logistic regression. *GAM*: modeling of the relationship with generalized additive models

In Fig. 2, the simulation results summarized four different approaches using the empirical covariate distribution and additionally the results for GLM and random forest using the covariate data drawn from the normal distribution are shown. The results from multivariate normal distribution with simple logistic regression event generation algorithm (GLM normal data) are interesting. In this approach, where the conditions where ideal meaning that all covariates, except the dichotomous sex, are normally distributed and the relationship is perfectly linear, there is no difference in ranking between the three measures. Consequently, the differences between the measures in other approaches could be explained by small deviations from the normal distribution of covariates and from the assumed relationship, which may not be perfectly linear or has been influenced by interactions. The mean ranks of the GLM method using normal covariate data differ strongly from the reference mean ranks except for MR-proADM using the Brier score difference and for hs-CRP using the IAUC or the *R* ^2^ difference. The GLM approaches with interactions (and quantile covariate data) and the GAM approach (using quantile covariate data) influence the results in a greater way than the covariate generating algorithm with GLM, but the reference mean ranks are still not reproduced. One possible explanation could be that the interactions and non-linear relationships were not considered in these approaches. The random forest approach (using quantile covariate data) addresses this points and leads to better results. For MR-proADM, the random forest approach yields in mean ranks comparable to the ranks in the reference, except of small deviations that remain under the simulation uncertainty. For NT-proBNP and hs-CRP with Brier score difference and IAUC, the deviations become larger due too weak effects and consequently larger uncertainty as well as for hs-CRP having the same reason. Only with Nagelkerke *R* ^2^ difference, the simulation fails to reproduce the reference mean ranks for the last two markers and yields in large deviations from the reference mean ranks that clearly exceed several fold the simulation uncertainty. In this setting, the GLM approach with quantile covariate data exhibits better performance. This may be due to the fact that the Nagelkerke *R* ^2^ is likelihood-based and therefore cannot detect model misspecification. Correspondingly, an oversimplified generating model may have no strong effect. If one compares the results of the random forest method using normally distributed covariate data with the results of the random forest method using the quantile covariate data, the same event generation algorithm is used, but the simulation of the covariate data differs. Here, the mean ranks of the two approaches are substantial different, using the covariate data drawn from the empirical distribution leads to better results in most cases. Only if one uses the IAUC or *R* ^2^ difference, the mean rank of MR-proADM does not differ between the two approaches. For the other event generating methods, this difference is also present and can be even stronger (results not shown). To sum things up, only the random forest approach using covariate data drawn from empirical distributions led to simulated data, where the ranking of the biomarkers approximates the reference ranking. The additional effort by using the data drawn from the empirical distributions is worthwhile as this, especially in the case of the RF as event generating algorithm, lead to remarkably better results. Furthermore, there were differences in precision of results of the measures for added value. The Brier score difference seems to have greater precision then the IAUC.

#### Absolute values criterion

**Fig. 3:**
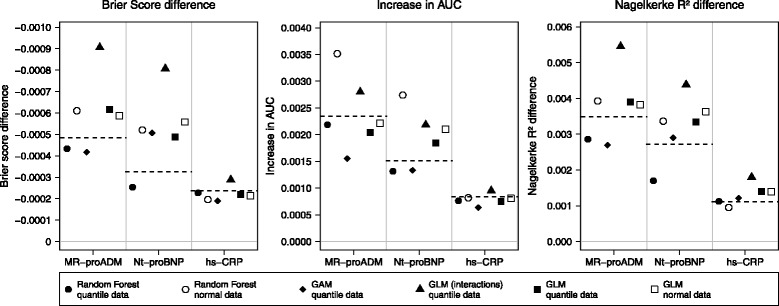
Simulated absolute values. Absolute values of differences were generated with 10000 simulation runs and with different models. *Dashed lines* represent the reference values, thus the differences between the values of the basic model and the values of the extended model in population data. *GLM* stands for generalized linear models, *GAM* for generalized additive models

In the following, the results are presented in terms of absolute values. Even if the simulation design was built up for stable ranking, it could be of interest to see the absolute values of the added value. These results are presented in Fig. 3. The results are compared to the reference effects in the population data. The incremental values are very small even for a strongly significant marker like MR-proADM. The basic Brier score of 0.163 was only reduced by 0.0005 with the strongest marker and by 0.00024 by the weak one (hs-CRP). The increase in AUC was somewhere between 0.00084 and 0.0024, which is apparently small compared to the basic AUC of 0.759. The same is also true for differences in *R* ^2^: The maximum of improvement is 0.0035 compared to 0.227 in the basic model. Like in the results regarding the mean ranks, the best approximation of the reference values was reached by random forest approach. The GLM model with interactions seems to overestimate the true improvement largely. The GAM model seems to be more accurate in some cases as GLM, but not overall. For the *R* ^2^ difference could not achieve a good approximation of the true values, comparable to the results from the ranking. To sum up, random forest approach were able to achieve good accuracy of simulated effects, at least for Brier score difference and the IAUC.

### Pilot sample size

**Fig. 4:**
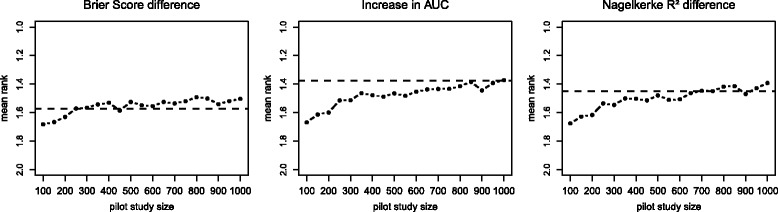
Pilot sample size investigation. Mean ranks for MR-proADM with random forest approach and different pilot sample sizes. For every step 1000 simulation runs were used. The *dashed line* represents the reference mean rank from population data

In the main simulation, the complete population sample as basis for the simulation was used. Consequently, it was a sample with 4519 observations as a source of information available. If one wants to use a pilot study or an interim analysis as the source of information, it is important to know at least approximately how large this subsample has to be to produce adequate results. A sample size over 1000 is unrealistic in real-life settings, particularly for a pilot study, where these observations are not used in the actual study. In terms of an interim analysis in a large cohort study with many thousand individuals, larger subsample size is conceivable. Even if the term pilot study is used in this work, these results will be as well valid for an interim analysis or other sources of raw data. In additional simulation, the needed sample size for a pilot study to cover the effects from our reference population was investigated. For this purpose, simulations with different sizes of pilot study samples, with which the artificial data was generated, were performed. Considering the results of the main simulation, the random forest approach with empirical distributions of covariate data was used for additional simulation. The pilot study samples were randomly drawn from our reference sample. Subsequently, the correlation structure, empirical distributions and the random forest model were generated based on this sample. The simulated data was built up with the original sample size of reference sample, so with 4519 observations. Only the results of the best marker MR-proADM is shown as example. These results are displayed in Fig. 4, where the mean rank for MR-proADM using all three measures and pilot sample size ranged from *n*=100 to *n*=1000 in 50th steps was calculated. For every step, 1000 simulation runs were used, which led to less precision compared to the main simulation. With Brier score difference, the reference value could be achieved with a sample size of 250. The IAUC had an acceptable, but not exact accordance with 250 observations. Here, the accordance only slowly improved with increasing sample size and reached a good value only at over 600 observations. This fits to the previous simulation results, where IAUC had a lesser precision than the Brier score difference. With *R* ^2^ difference, the sample size of 350 was needed for a good accordance with reference values. The interpretation of results with *R* ^2^ difference is difficult, because it fails to reproduce all effects properly and showed sufficient performance only for the strongest marker. It should be noted that these results cannot be generalized, because the needed sample size for a pilot study is strongly dependent on the effect size of the marker, the clinical outcome and, as we have shown, from the choice of the measure. But even for weak effects in the application example a sample size of about 250 seems to be sufficient, which should be a feasible sample size for a pilot study.

## Discussion

In this work we investigated the necessary tools to perform simulation of realistic data with complex structure, e.g. to plan a new study for biomarker comparison or for other subject matter questions corresponding to a multivariable analysis. Except for one specific performance measure (the Nagelkerke *R* ^2^), our results indicate a good simulation should be based on individual level pilot data, as the detailed information required for an adequate simulation cannot be extracted from typical aggregate results given in prior publications. Artificial data from theoretical distributions fail to represent the real situation properly and can lead to wrong results. Even small violations of assumptions, which are often negligible in other situations, could be essential for simulation results. Based on a comparison of four different approaches for generating the outcome, the random forest approach (using covariate data drawn from the empirical distribution) seemed to be the most successful. These results are also valid if one takes into account the variation of the rank, which was comparable between the different approaches. Unfortunately, this is the most complex model considered and as all machine learning methods it requires more data [42]. It leads us to the conclusion that non-linear relationship and interaction are playing an important role. Although our reference sample had no clear non-linear relationships, there may be small but nevertheless relevant deviations from linearity. For the analysis of a sample it is common to use a linear model even for a relationship that may be not perfectly linear in order to avoid overfitting, but for a simulation setting is seems to be rather crucial to also consider small deviations from linearity. As the basic model was extended only with one marker at a time, interactions between biomarkers would often be missed in an analysis, but they are playing an important role for the marker ranking in simulations. We think the appropriate modeling of the relationship is a more important aspect than the distribution of covariate data, as long as the correlation structure was adequately reflected. This last part is achieved in a straightforward manner by using covariate data drawn from the empirical distribution, although this requires more information. Nevertheless, we think that this effort is worthwhile as it results in better simulation results and does not need to be adapted if the distribution is non-normal. Additionally, the random forest approach does also need the information on the individual level and thus using the empirical distribution is no additional expenditure in this case. One known disadvantage of random forest approach is the black box nature of the model, but as our aim is to perform a data based simulation, there is no need for a good interpretability of generating model. The model must only adequately reflect the data structure and deliver realistic simulated outcomes. Another aspect to be considered when using the random forest approach is the appropriate choice of tuning parameters, such as the tree size. The difficulty is to choose these parameters in a way that ensures sufficient degrees of freedom, but avoids the overfitting at the same time. The tree size determines the interaction order of the model, so trees with only one split, so called stumps, result in a model without interactions. Each additional split increases the order of possible interactions. This parameter should not be too small; otherwise the model fails to reproduce the interactions in the data. In case of simulations, the choice of adequate tree size is not very critical as long as the model has sufficient degrees of freedom, because a certain amount of overfitting is even desirable. The model must not be generalizable for new data, on the contrary, it must simulate the data with their special characteristics such as random noise as well as possible. Consequently, the pruning of the trees might not be as important in this case. Instead of recommending an added value measure, we recommend to include the choice of the added value measure into the planning phase. For example, a better separation between the markers was possible when using IAUC, but the Brier score difference had a better precision in results. Only the Nagelkerke *R* ^2^ difference is not recommended as the results could not be recovered well within a simulation study. Another aspect that should be taken into account when choosing the measure is the sample size of a pilot study or interim sample study. With the right choice of the measure, the required sample size could be reduced. From this point of view, the Brier score difference can be recommended. We used the improvement of prediction in nested models with adding a new single marker, but this concept could be used as well for improvement with several new markers simultaneously. This could be important when investigating weak markers, like genetic SNP markers for example [43], where interaction between markers could play an even more important role.

In addition to the scenarios investigated here, other data analysis scenarios might be considered, e.g. a comparison of two sets of markers or a set of markers with one single marker. We expect that our results also transfer to such other multivariable settings to some extent, as the strategy presented here ensures that the correlation structure with exact covariate distribution is considered as well as the relationship to event with interactions and non-linearity. Naturally, our results may strongly depend on the specific data source, the Gutenberg Health Study (GHS), and the specific markers and outcome considered in our investigation, but we expect that the level of complexity seen there is not unusual and needs to be anticipated when setting up a large cohort study.

## Conclusions

Generalizing from the present results, we would not recommend potentially oversimplified regression models for representing the relationship between markers and the outcome when simulating complex data. It seems that more flexible approaches, such as random forest, may be more appropriate for adequate simulation of complex multivariate data. This better takes into account important aspects such as non-linear relationships or interactions and therefore provides more adequate results. Yet, these methods require information on individual level and thereby a pilot study or other preliminary data sources. Additionally, the results of the simulation emphasize that some not readily apparent properties of the underlying data structure can affect (the performance of) marker identification. This also is an important lesson for other situations where realistic data structure needs to be simulated.
